# The British Medical Association in Toronto

**Published:** 1906-09-29

**Authors:** 


### THE BRITISH MEDICAL ASSOCIATION AT TORONTO.

By a Canadian Correspondent.

The British Medical Association meeting is over, and the 2,000 medical men and women who took part in it are scattering by various routes more or less direct to the ends of the earth from which they came—England, Scotland, Ireland, Germany, France, Belgium, Egypt, South Africa, India, Australia, New Zealand, the West Indies, the United States, and all the different Provinces of Canada itself. All who have taken part in the work of the different sections are agreed that, from a business point of view, the meeting has been a great success. Never have more able and illuminating papers been read to larger or more appreciative audiences. The numerous sections were all well attended, and the attendance was well sustained until the end of the meeting. Much new food for thought and new matter for patient investigation were provided, and all who took the trouble to follow the work of the different sections were able to form a fairly good idea as to the lines upon which medical and surgical progress is likely to run for the next few years.

Leaving aside the purely professional aspect of the meeting, what is likely to be the result of the coming together here in this new land of so many men and women from all quarters of the globe; and what impressions of our country and our ways will our visitors take home? Inevitably we must have learned to know one another better. The British and Canadian medical schools have been brought nearer one another; and now, when our young men come over to you, they will come with the confidence that they are no longer strangers from an unknown land.

We hope that under an aggressive, unceremonious, self-satisfied exterior you will have discovered amongst our professional men a profound admiration for, and a humble desire to learn from, the intellectual lights of the older and in many ways more favourably situated lands; a desire clearly shown to exist by the large proportional number of men out of our small population who every year put themselves to the expense and loss of practice of a long course of study in the British and Continental schools, where, we are glad to hear, they are making for themselves a reputation as the backbone of the post-graduate classes.

Visitors from the Old Country will, no doubt, have discovered that here in Ontario, and more or less all through the English-speaking provinces of Canada, our people approximate in their manners and customs, in their methods of trade, and in their language to our neighbours to the South. This can scarcely be wondered at when it is realised that out of a nation of about 8,000,000 people over 2,000,000 are at present holding, or during some period of their lives have held, positions in the United States; that there is a constant interchange of business men between the two countries, and that all the summer long our hotels and lodging-houses are filled to overflowing with American holiday-makers; and, last but not least, that, for some unknown cause, all American newspapers and periodicals are allowed by our Government to come into this country free, and, from an equally unknown and malign cause, the British Government, by its postal regulations, practically shuts out the whole of the English magazines from Canada. It may have surprised those who have observed us closely that, "in spite of all temptations to belong to other nations," and although our customs, and even our ideals, are becoming more and more closely related to those of the United States, we are every day developing a more strongly national Canadian feeling, and showing less and less tendency to throw in our lot with our neighbours.

You have been to our dinners, our lunches, and our garden-parties. You have seen our universities and schools, our public buildings and our unpainted shacks, our private houses and our Chinese laundries. We have shown you our great possessions and our monuments of commercial enterprise; and, with youthful pride, told you time and time again until you are weary of it that we are a great and glorious nation. Forgive us our youthful sins! Those of us who have been mellowed by a more or less intimate acquaintance with older civilisations know full well that Niagara Falls Electric Power Works, the vast lakes, the endless plains, the glorious Rocky Mountains, and the mighty forests, with their wealth of timber and precious metals, do not necessarily make a great nation. The millions who are coming into our land must in the near future build up a large nation; and we pray that they will develop such ideas of righteousness and of justice, of beauty and of truth, as will make it truly great.

When our ardent enthusiasts were giving you the benefit of their overflowing pride we were painfully conscious that you were inwardly wishing we would mend our manners and our roads, provide you better service in the hotels, do something to justify the reputation of Canada for a cold, dry, bracing climate and get rid of the spell of oppressing moist heat under which you, and even the gentlemen from India, were suffering, and rescue your checked baggage from the depths of you knew not where. Our excuse for our roads is that they are of recent origin, and we have had many to make. The red man was unlike the Roman—he had no use for roads. His canoe was his chariot, and for snowshoeing not even a track is required. The spell of weather was like the B.M.A. meeting, something that many of us had never had experience of before; and should any visitors be still with us to-day they will be rejoicing in a temperature of 62°, with the prospect of an almost frosty night, instead of suffering under a temperature of 92°. Good service, such as English people are accustomed to in even small hotels in the old lands, cannot be got in the land of freedom and equality. It may shock our visitors to see the waiter who after dinner hands them their cigars put one into his own mouth and join them in their smoke; but this is the spirit of the land. That waiter is not doomed to perpetual flunkeyism, but is on the way to better things. It was unfortunate that our vaunted checked baggage system should have so hopelessly broken down, but an unprecedented stress was put upon our railway officials at a time when thousands of our young men were away in the harvest-fields of the West.

Then may we hope that as a result of this great meeting England will know Canada better; that letters will no longer arrive addressed to us "Toronto, Canada, U.S.A.," and official documents turn up addressed to the "British Consul at Toronto," as constantly happens now; that you will give all the intelligent sympathy and help you can to a daughter nation, bound to be large, hoping and striving to be great? Send out to us your men of literature, of science, of philosophy, and of art. Keep your political firebrands at home. Let us exchange freely knowledge and ideas, that your ideals may grow to be ours, and our ideals yours, and so Canada be for ever one with the Old Land.

